# Altered environment and risk of malaria outbreak in South Andaman, Andaman & Nicobar Islands, India affected by tsunami disaster

**DOI:** 10.1186/1475-2875-4-32

**Published:** 2005-07-20

**Authors:** Kaliannagoun Krishnamoorthy, Purushothaman Jambulingam, R Natarajan, AN Shriram, Pradeep K Das, SC Sehgal

**Affiliations:** 1 Vector Control Research Centre, Indira Nagar, Pondicherry, India; 2 Regional Medical Centre, Port Blair, Andaman & Nicobar Islands, India

## Abstract

**Background:**

Pools of salt water and puddles created by giant waves from the sea due to the tsunami that occurred on 26^th ^December 2004 would facilitate increased breeding of brackish water malaria vector, *Anopheles sundaicus*. Land uplifts in North Andaman and subsidence in South Andaman have been reported and subsidence may lead to environmental disturbances and vector proliferation. This warrants a situation analysis and vector surveillance in the tsunami hit areas endemic for malaria transmitted by brackish water mosquito, *An. sundaicus *to predict the risk of outbreak.

**Methods:**

An extensive survey was carried out in the tsunami-affected areas in Andaman district of the Andaman and Nicobar Islands, India to assess the extent of breeding of malaria vectors in the habitats created by seawater flooding. Types of habitats in relation to source of seawater inundation and frequency were identified. The salinity of the water samples and the mosquito species present in the larval samples collected from these habitats were recorded. The malaria situation in the area was also analysed.

**Results:**

South Andaman, covering Port Blair and Ferrargunj sub districts, is still under the recurring phenomenon of seawater intrusion either directly from the sea or through a network of creeks. Both daily cycles of high tides and periodical spring tides continue to cause flooding. Low-lying paddy fields and fallow land, with a salinity ranging from 3,000 to 42,505 ppm, were found to support profuse breeding of *An. sundaicus*, the local malaria vector, and *Anopheles subpictus*, a vector implicated elsewhere. This area is endemic for both vivax and falciparum malaria. Malaria slide positivity rate has started increasing during post-tsunami period, which can be considered as an indication of risk of malaria outbreak.

**Conclusion:**

Paddy fields and fallow land with freshwater, hitherto not considered as potential sites for *An. sundaicus*, are now major breeding sites due to saline water. Consequently, there is a risk of vector abundance with enhanced malaria transmission potential, due to the vastness of these tsunami-created breeding grounds and likelihood of them becoming permanent due to continued flooding in view of land subsidence. The close proximity of the houses and paucity of cattle may lead to a higher degree of man/vector contact causing a threat of malaria outbreak in this densely populated area. Measures to prevent the possible outbreak of malaria in this tsunami-affected area are discussed.

## Background

Close monitoring of public health problems in disaster-hit areas is warranted to assess the risk of disease outbreaks, including vector borne diseases. In this context, WHO has released a technical note on malaria risk and its control in areas affected by the tsunami disaster [1]. Updates of the tsunami situation are also available in weekly reports covering all the affected areas [2]. A review on the malaria situation preceding the tsunami in Sri Lanka outlined the key issues to be considered [3]. Observations made in one of the tsunami-hit areas in the Andaman district of the Andaman & Nicobar Islands, India, which had hitherto been a low endemic area for malaria with no major outbreaks of communicable diseases, are presented here. There is a need for extreme vigilance in view of vulnerability of these densely populated areas and extent of breeding grounds of the local vector, *Anopheles sundaicus *created by the tsunami.

Andaman and Nicobar islands (92° to 94° East and 6° to 14° North) is an archipelago of 555 islands/islets, stretching over 700 kms from north to south, in the Bay of Bengal. There are 38 inhabited islands with a population of approximately 356,000. This is a Union Territory under the governance of the Andaman & Nicobar Administration. Many creeks traverse the mainland with tributaries of varying lengths and during high tide, water from the sea penetrates creating swamps and marshes, which nurture the thriving mangrove. The topography of the islands is hilly and abounds in evergreen forests. The climate is tropical hot (24° to 30°C temperature) and humid (mean relative humidity of 78.5%) with abundant rainfall (normal annual rainfall of 3,180 mm), supporting a luxuriant and rich vegetation. Both northeast (November to April) and southwest monsoons (May to October) are active, though the latter is weak. About 92% of the total geographical area of 8,248 km^2 ^is forest covered.

There are two districts in Andaman & Nicobar Islands. Andaman district is largest with a surface area of 6,408 km^2 ^and population of approximately 314,000 in 25 inhabited islands [4]. While there are many perennial rivers in Nicobar district, only perennial streams are present in Andaman district. Andaman district has two subdivisions; South Andaman and Mayabunder (Middle and North Andaman). Port Blair, the capital of these islands is situated in South Andaman. Andaman and Nicobar Islands are an important tourism center attracting both national and international visitors in large numbers, throughout the year. Coconut, areca nut, banana, and rice are the major crops grown in addition to forest products. Rain-fed paddy cultivation is done between October and January during the northeasterly monsoon season. Paddy fields cover about 10,517 hectares with an annual production of 46,276 metric tons.

Malaria has been endemic in the Andaman and Nicobar Islands for nearly a century and *An. sundaicus *is the incriminated vector [8]. During 1992–2004, the Annual Parasitic Incidence (API) ranged from 1.42 to 4.5 per thousand. Till now, the Andaman district (API = 0.64) remained of low endemicity when compared to the Nicobar district (API = 8) [1], with about 18% of the total cases due to *Plasmodium falciparum *in 2004.

The earthquake under the sea, which caused the tsunami on 26 December 2004, caused extensive damage in the Andaman & Nicobar Islands, which are classified as seismic zone 5, indicating high level of risk due to earthquakes. The extent of environmental disturbance caused by the tsunamis is evident from the post-tsunami reports. Run-up level of seawater (above normal tide or mean sea level) during tsunami varied from 1.4 to 7.0 m in selected locations and the distance penetration from the coast ranged from 100 to 250 m [5]. Most of the northern Andaman islands were uplifted, but subsidence of 1 – 1.5 m was reported from Port Blair and Ross Island (South Andaman) [6]. Little Andaman in Andaman district was uplifted by 1 m. As many as 215 aftershocks have occurred so far with varying magnitudes ranging between 5 and 7 on the Richter scale. Thirty islands with a population of 0.18 million are affected by the tsunami and coastal flooding. According to the latest information (12^th ^March 2005) from the Ministry of Home Affairs, human lives lost was 1,395 due to the tsunami, with 5,764 still missing. The damage to human lives and belongings was highest in Nicobar, less in Andaman district, with a reported toll of 79. There are 44,183 persons in 135 relief camps. The entire population from six islands in the Nicobar districts has been evacuated.

## Methods

**Figure 1:**
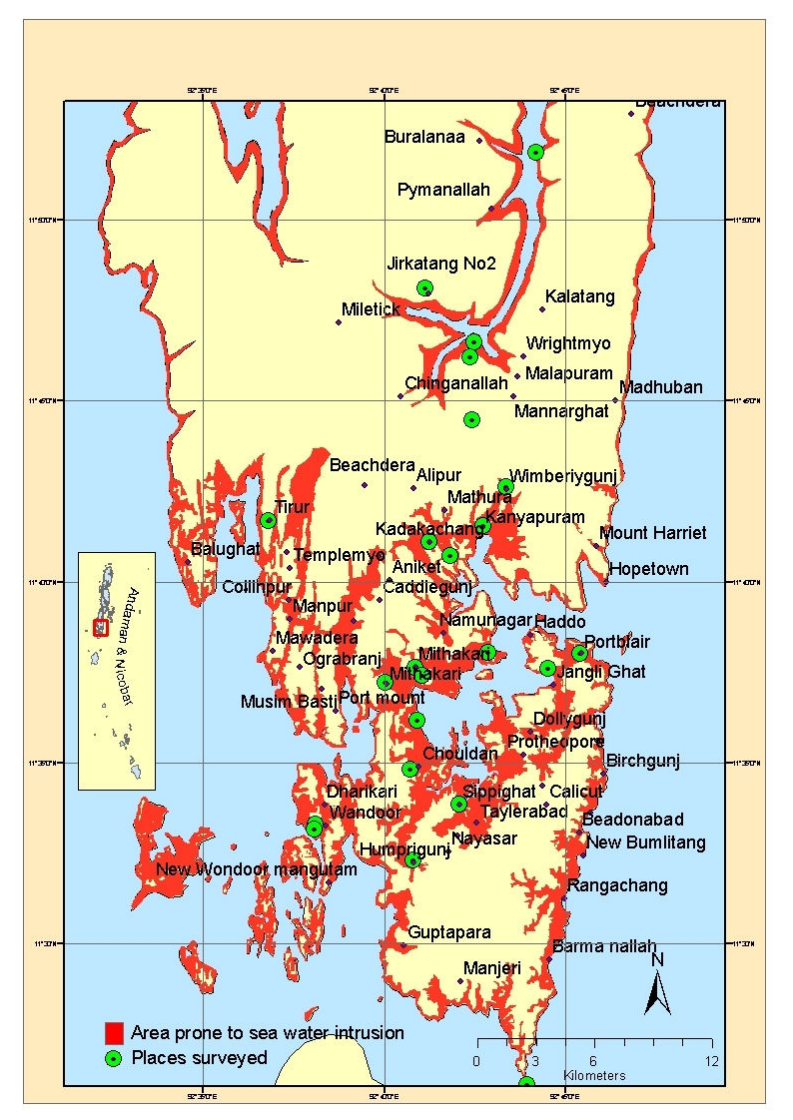
Map showing the location of villages and areas vulnerable for seawater intrusion from the sea or creeks in South Andaman. Courtesy: Institute of Ocean Management, Anna University, Chennai.

An extensive survey was carried out in Andaman district covering both the South Andaman and Mayarbunder subdivisions during March 2005, two months after the disaster. The objective was to assess the environmental risk and consequent risk of malaria due to seawater inundation following the tsunami. Two sub-districts (Port Blair and Ferrargunj), with a population of approximately 208,000 in South Andaman, are still affected by seawater inundation. There are two major creeks, Wright Myo in the middle and Shoal Bay in the north. 22 locations were visited and co-ordinates were recorded using Geographic Positioning System (GPS) for preparing digital map. Institute of Ocean Management, Anna University, Chennai prepared a vulnerability map of South Andaman from Shuttle Radar Topography Mission (SRTM) imagery and identified the areas within 0 and 20 m as most vulnerable for seawater intrusion based on elevation. This map, posted elsewhere  was used as the base map and the locations of the survey sites were marked (Fig. 1). Details of the source and frequency of seawater intrusion, environmental risk in terms of altered ecological parameters such as salinity, damage to the crops, type, and extent of habitats flooded were collected. Mosquito breeding was checked in different habitats. Water samples along with larval samples were collected from these habitats. Salinity, pH, mosquito species composition, and their immature density were recorded. Density of anopheline species was categorized based on average number of larvae per dip into level 1 (1–10), 2 (11–100) and 3 (above 100). Larvae, collected from the selected habitats were reared to adults for species identification. Both larval and adult characters were used for species confirmation, following the key of Christophers [7]. Water samples collected from these surveyed habitats were analysed for salinity using titration method.

## Results

**Figure 2:**
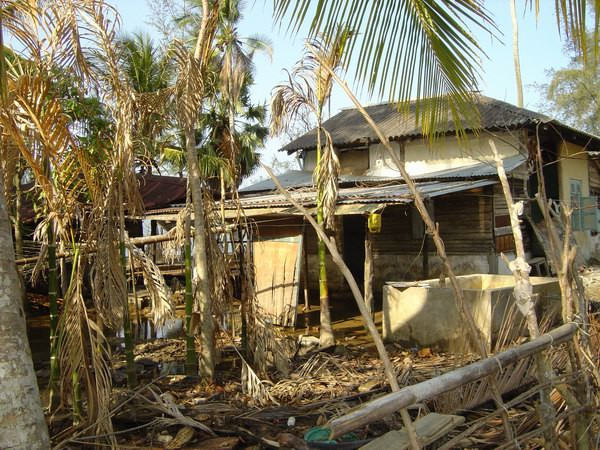
Photograph showing the destruction of areca nut and coconut grooves due to seawater inundation.

**Figure 3:**
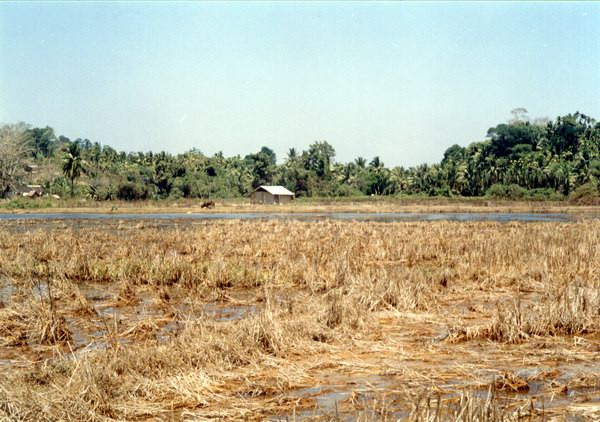
Photograph showing the paddy field flooded with seawater.

Seawater inundation was observed to recur both directly from the sea (Chouldari and Wandoor) and through the network of creeks (Sippighat in Port Blair, Bambooflat, and Winberlygunj). The areas inundated have been categorized into three types: (i) areas where seawater intrusion occurred once during the tsunami leaving sheets/pools of stagnant water which are gradually drying up, (ii) areas where intrusion of seawater recurs periodically at spring tide depending on the lunar cycle and (iii) areas where inundation takes place twice daily due to high tides. Damage to the areca nut (Fig. 2) and coconut grooves, banana plantations and paddy cultivation (Fig. 3) was extensive in the seawater-flooded areas. The paddy crop in about 500 hectares was totally destroyed due to seawater ingression before harvest and due compensation was paid as a relief measure. In a few pockets (Sippighat), even mangroves were affected and this could be due to sudden rise in salinity. During high tide, even roads get flooded, affecting movement of vehicles in some areas. Areas associated with Wright Myo creek in between Port Blair and Bambooflat are extensively affected. Inundation of inland low-lying areas during high tide has become a concern to the local population as their houses are marooned in seawater. Shoal Bay in the north traverses through reserve forest, which is elevated and not prone to seawater intrusion. Similarly, Little Andaman, which is a part of South Andaman is currently free from seawater intrusion, though there are waterlogged areas created by the tsunami waves. Middle and North Andaman are elevated areas within short distance from the seacoast. Due to cliff and forests, there is no area prone to seawater inundation. Apart from seawater logging in the paddy fields and fallow lands, intrusion of seawater resulted in the flooding of freshwater ponds and canals and creation of a number of swamp pools and stagnant pools.

A total of 46 habitats were surveyed and 38 of them were either created by or under the influence seawater intrusion. The location of the survey and the type of habitats in relation to frequency and source of seawater intrusion are given in Table 1. (see Additional_file_1.xls). Examination of these habitats showed the breeding of anopheline mosquitoes in 35 habitats. *An. sundaicus *was found in at least 19 (54%) of these habitats. Habitats supporting breeding of this vector species were widespread in South Andaman. Vector breeding was observed in 77% of the habitats under the influence of spring tide while it was 46% in habitats flooded once during tsunami and 25% in habitats with daily seawater intrusion during high tide. The larval density was dense (10–100 larvae/dip) in habitats prone to spring tide while it was sparse (1–10 larvae/dip) in habitats under continuous influence of tidal waves (Table 1). These habitats were also found to support the breeding of *Anopheles subpictus *(71% of the positive habitats) and *Anopheles vagus*, vectors implicated elsewhere. The salinity in the habitats under the influence of seawater intrusion was found to range between 3,000 and 42,505 ppm (parts per million). The salinity in the habitats such as streams, ponds and stream pools, swamps was less than 1,000 ppm. The mean salinity level was 30,668 and 28,026 ppm respectively in habitats under the influence of spring tide and every day high tide. Habitats flooded with seawater once during tsunami were recorded with a mean salinity level of 18,250 ppm. Breeding of *An. sundaicus *was found in habitats with salinity ranging between 3,331 and 42,505, with a mean of 25,150 ppm. pH was found to range between 6.0 and 8.7 in different habitats.

**Figure 4:**
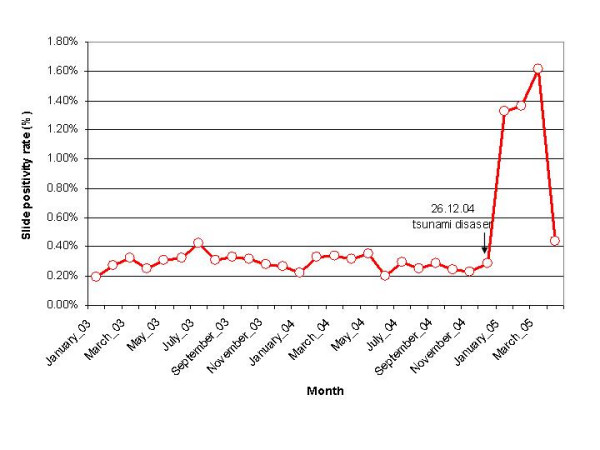
Monthly Slide positivity rate for malaria in Andaman district.

Temporal analysis of malaria data (active and passive surveillance of fever cases) from the Directorate of Health Services showed that Slide Positivity Rate (SPR) for malaria in Andaman district fluctuated between 0.2 and 0.4% in different months during 2003 and 2004. There was about 5–6 fold increase in SPR during the months following tsunami (Figure 4) although the blood smear examination rate remained almost at the same level of around 3% during post-tsunami months (January to March 2005). A similar trend was evident in the Nicobar district and the SPR was 10.59% during the post-tsunami while it was 0.75% during 2004.

All the five PHCs in the areas prone for seawater intrusion were endemic for malaria. The SPR ranged from 0.12 and 0.39% in these PHC during 2003 and 2004 (pre-tsunami period). *P. falciparum *was also recorded with an overall Slide Falciparum Rate (SFR) of 0.05%. During the months of January and February 2005 (post-tsunami), the SPR in Tusnabad PHC, which covers the most vulnerable area for seawater intrusion, was 1.52 and 1.26 respectively while it was 0.14% during 2004.

## Discussion

Andaman and Nicobar districts have been endemic for malaria for nearly a century. Both vivax and falciparum malaria occur, with about 18% of the total cases due to *P. falciparum *in 2004. *An. sundaicus *continues to the most predominant species transmitting malaria in Nicobar group of islands [15,16,19] although it has become very scarce in many peripheral areas of distribution in India [9,14]. Relatively, Andaman district was low endemic and *An. sundaicus *is reported to be less abundant [13]. In Nicobar group of island, this species breeds mainly in lagoons and creeks [15]. There are many perennial rivers in Nicobar district and the lagoons at the river mouth can support profuse breeding of this vector species. Only perennial streams are present in Andaman district. Thus, greater extent of potential vector breeding habitats and high vector abundance can be attributed to higher prevalence of malaria in Nicobar district compared to Andaman district.

During the post-tsunami months, malaria showed an increase in both the districts. South Andaman, which was low endemic prior to tsunami, showed an increase in SPR for malaria to an extent of 5–6 folds during post-tsunami. It is an indicative of potential risk of malaria outbreak. It would also be a matter of concern in view of the reported poor response of *P. falciparum *to chloroquine in Little Andaman [20]. Such a situation is a consequence of land subsidence caused by tsunami with extremely favourable breeding habitats, which were non-existing prior to tsunami. *An. sundaicus *is known as a brackish-water mosquito, though reported to breed in a wide range of habitats [12], possessing the ability to adapt to available sites. In addition to its original breeding grounds of creeks and streams [13] in South Andaman, now its breeding has been extended to paddy fields and fallow lands flooded with seawater. Paddy fields and fallow lands with fresh water hitherto considered, as least potential sites for *An. sundaicus *are now a major breeding habitat due to saline water. Destroyed paddy fields are perhaps the first record of major breeding source for *An. sundaicus*.

Organic pollution in the form of putrefying masses of vegetation is reported to provide very favourable conditions for breeding of *An. sundaicus *[17]. A similar situation is indicated in South Andaman due to decaying paddy plants. *An. subpictus *and *An. vagus*, implicated vectors elsewhere [10,11] were also recorded in these habitats, but their role in malaria transmission is not known. Profuse breeding of *Aedes verrallina *and *Culex sitiens *in seawater-flooded paddy fields and stagnant pools add to the existing mosquito biting menace to the locals. Rain following the onset of monsoon in May/June may worsen the situation by increasing the breeding surface area manifold. As this species is known to tolerate salinity from freshwater to much greater concentration than seawater [12], approaching monsoon may aggravate the situation due to likelihood of further increase in breeding surface area, in view of recurring nature of seawater intrusion. The chances of turning back to pre-tsunami situation seem to be limited, due to land subsidence and related permanent environmental alterations. However, stagnant pools created during tsunami with one time flooding are temporary in nature and the salt may get depleted due to rainfall in course of time.

*An. sundaicus *is a species complex with four siblings and sibling species D has been identified from the Car Nicobar islands [24]. The adult behaviour is heterogeneous with differential behaviour in different geographical regions, indicating species diversity. This species is zoophagic (prefer to feed more on animals) [18], exophilic (prefer to rest outdoors) and exophagic (prefer to feed outdoors) [25] in Nicobar district. This is an opportunistic feeder and exhibits a peak of biting activity from 8.00 PM to 3.00 AM. Due to paucity of cattle (man to cattle ratio is 1:0.29) and the proximity of houses to the newly created, potential vector-breeding habitats in South Andaman, which is to-day the most densely populated area (70 per km^2 ^against an average of 43 per km^2^), increased risk of man/vector contact exists. There was evidence of local epidemics due to *An. sundaicus *in Orissa from 1930–1940 [21] and Calcutta in 1936 [22] and other Southeast Asian countries as well [23].

During the 1950s, control of this vector was based on the application of DDT inside houses in many Southeast Asian countries [26]. In Indonesia, the control efforts were focused on environmental alteration of breeding sites by drainage [27]. Use of larvivorus fishes, biocides, shading, and elimination of vegetation were also tried to control the breeding [12]. However, these methods require continuous monitoring and follow-up. Antilarval measures with weekly application of Baytex/abate and indoor residual spray with DDT were the methods of vector control against malaria during the pre-tsunami period in Andaman and Nicobar islands. Early case detection and treatment through passive and active surveillance was also carried out. Following tsunami, these measures were intensified in addition to measures such as fogging with malathion, release of larvivorous fishes and environmental measures wherever possible. Insecticide impregnated bed nets were distributed in the disaster relief camps. During the pre-DDT era, sluice gates were constructed at the outlets of streams with automatic wooden gates, which were intended to let out the accumulated rainwater at low tide and automatically close as the tide rises [8]. By preventing the intrusion of seawater, the freshwater marsh/swamp becomes unsuitable for *An. sundaicus *to breed. Difficulties in continued maintenance had become serious. These gates were destroyed following the tsunami. Reports on the beneficial effect of this measure on malaria are however not available. Locals around the Wright Myo creek have already approached the Andaman & Nicobar Administration to get these sluice gates repaired to prevent seawater intrusion, protect their houses, and prevent the inundation of paddy fields. Though it may be possible to restore the functionality by repairing the damaged sluice gates, continued maintenance may be an important issue. If the entire area is targeted, as there are many tributaries of this creek, the construction of sluice gates will not only be expensive but also affect the current wetland mangroves, which accounts for about one fifth of the 4,827 km^2 ^of area under mangrove vegetation in India. In view of land subsidence [6], construction of sluice gate alone may not be adequate to prevent seawater inundation to bring back pre-tsunami state. Earth embankments may be useful to prevent seawater intrusion where the entry point is narrow. However, these measures may not offer any immediate solution to the problem of malaria transmission.

Promotion of the use of personal protection measures, with long-lasting insecticide impregnated bed nets, can be the method of choice to prevent malaria in South Andman. This could offer protection against menacing mosquitoes also that bite at night. Currently such nets have been distributed in Nicobar district but in Andaman district, its distribution was limited to camps. Indoor residual spray with insecticides is a community control measure and therefore high coverage is essential for control. However, this measure will be wasteful against this outdoor resting vector and antilarval measures will not be practicable in view of the vastness of the breeding grounds. Being a strong flier with a flight range of 9.6 km [17], the area of operation to control the breeding of this mosquito will be extensive if larval/environmental management methods are resorted to.

Malaria surveillance, through passive and active case detection in all the five PHCs in Port Blair and Ferrargunj, should be strengthened to prevent the incidence and spread of malaria. Basic entomological studies on vector abundance, species complex, and behaviour are necessary to detect changes related to the rapidly changing environment due to a natural disaster like the tsunami in South Andaman. In addition, long-term observation on water levels in these areas may be useful to understand the impact of land subsidence in this area.

## Conclusion

This paper provides the results of an extensive survey carried out on the risk of vector proliferation and consequent risk of malaria outbreak in the tsunami-affected areas in the Andaman district of the Andaman and Nicobar Islands, India. Environmental damage with altered ecological factors was observed in South Andaman, covering Port Blair and Ferrargunj sub-districts. With the evidence of land subsidence, this area with a population of approximately 208,000 is still suffering the recurring phenomenon of seawater intrusion either directly from the sea or through the network of creeks. Both daily cycles of high tides and periodical spring tides continue to cause flooding. Low-lying paddy (destroyed) fields and fallow land habitats with freshwater, hitherto considered the least potential sites for *An. sundaicus*, are now a major breeding source due to saline water, with the salinity ranging from 3.000 to 42,505 ppm. The extent of these tsunami-influenced breeding grounds and the likelihood of them becoming permanent due to continued flooding are indicative of vector abundance. Both vivax and falciparum malaria occurred in this area but the incidence was low. Proximity of houses to flooded paddy fields and paucity of cattle may lead to higher degree of man/vector contact causing a threat of malaria outbreak in this densely populated and low endemic area. Temporal analysis of malaria cases in Andaman has shown an increasing trend following tsunami. There is an urgent need for a long-term and systematic monitoring of environmental risk and vector surveillance. Considering the man-biting and daytime resting behaviour of *An. sundaicus*, promotion of the use of personal protection measures with long-lasting insecticide-treated bed nets and intensified Early Detection and Prompt Treatment (EDPT) of malaria cases are recommended to prevent the possible outbreak of malaria.

## Conflicts of interest statement

The author(s) declare that they have no competing interests.

## Authors' contributions

KK carried out situation analysis, data analysis and report writing. PJ collected and analysed data on environmental parameters. RN recorded mosquito species composition. ANS collected mosquito and water samples for analysis. PKD performed epidemiological analysis and critical reviewing of the manuscript. SCS provided technical and administrative support. All authors reviewed the report and approved the final manuscript.

## Supplementary Material

Additional File 1 This file is in excel format containing table 1. Click here for file
